# Advanced Hybrid Polysaccharide—Lipid Nanocarriers for Bioactivity Improvement of Phytochemicals from *Centella asiatica* and *Hypericum perforatum*

**DOI:** 10.3390/pharmaceutics18010048

**Published:** 2025-12-30

**Authors:** Ioana Lăcătusu, Mihaela Bacalum, Diana Lavinia Stan, Ovidiu-Cristian Oprea, Mihaela Neagu, Georgeta Alexandru, Mihaela Prisacari, Nicoleta Badea

**Affiliations:** 1 Faculty of Chemical Engineering and Biotechnology, National University of Science and Technology Politehnica Bucharest, Polizu No 1, 011061 Bucharest, Romania; ioana.lacatusu@upb.ro (I.L.); ovidiu.oprea@upb.ro (O.-C.O.); mihaela.prisacari@stud.chimie.upb.ro (M.P.); 2 Department of Life and Environmental Physics, Horia Hulubei National Institute of Physics and Nuclear Engineering, Reactorului Street No. 30, 077125 Magurele, Romania; bmihaela@nipne.ro (M.B.); diana.stan@nipne.ro (D.L.S.); 3 Academy of Romanian Scientists, 3 Ilfov St., 050044 Bucharest, Romania; 4 S.C. Hofigal Export Import SA, Intrarea Serelor No. 2, 042124 Bucharest, Romania; mihaela.neagu@hofigal.eu (M.N.); gabi.alexandru@hofigal.eu (G.A.)

**Keywords:** *Centella asiatica*, *Hypericum perforatum*, lipid nanocarriers, hyaluronic acid, wound healing

## Abstract

**Background/Objectives:** Phytochemicals are known to be active contributors to a healthy life, providing valuable wound healing benefits. **Methods:** This research took an innovative approach that successfully overcame the bioavailability limits of herbal extracts, by entrapping *CentellaA* with *HypericumP* in nanostructured lipid carriers (NLCs) and hybrid hyaluronic acid (HA-NLCs) as valuable formulations with enhanced bioactivity. **Results:** NLCs and HA-NLCs showed excellent entrapping efficiency values for *CentellaA* and *HypericumP* ranging from 89.5 to 95.3%. Co-entrapping of *CentellaA:HypericumP* in a weight ratio of 4:1 and 2:1 led to diameters of 221.4 ± 2.08 nm for NLC-*CentellaA-HypericumP* and 220.3 ± 1.74 nm for hybrid HA-NLC-*CentellaA-HypericumP*. The bimodal calorimetric profile of NLCs contributed to a lower degree of lipid core structural organization. HA-NLC-*CentellaA* showed the safest biocompatibility behavior with BJ skin cells. **Conclusions:** The cells treated with NLC-*CentellaA* exhibited a favorable scratch wound closure and promoted the fastest BJ cell migration. NLC- and HA-NLC herbal extracts remodeled the cytoskeleton of BJ fibroblast cells. The morphological fluorescence changes revealed that the fibroblast cells retained intact their cytoskeleton, characteristic of a viable cell with no obvious stress. An active motility of cells treated with NLCs in the wound area was detected, indicating strong pro-migratory properties; e.g., for NLC-*CentellaA*, the wound was almost closed after 30 h. Designing NLCs with HA adaptability to reinforce the skin wound healing action represents a desired step for the development of herbal products that meets the challenge of combining the benefits of phytochemicals and nanotechnology to create value-added herbal products.

## 1. Introduction

Pharmacological and lifestyle changes are key strategies for maintaining a healthy lifespan. Phytochemicals such as polyphenols, triterpenoids, flavonoids, phenolic acids, and tannins, are known to extend longevity and improve quality of life [1]. *Centella asiatica* (*CentellaA*) and *Hypericum perforatum* (*HypericumP*) extracts have recognized bioactivity, exhibiting notable therapeutic potential such as antidiabetic, antimicrobial, anti-inflammatory, anticancer, neuroprotective, antioxidant, and wound healing activities [2,3].

*Centella asiatica* has a high content of triterpenes (e.g., Asiatic acid, madecassic acid, asiaticoside, madecassoside), polyphenols (such as flavonoids, phenolic acids, anthocyanins, proanthocyanidins, etc.), phytosterols, amino acids, and sugars, which contribute to its broader medicinal applications [4]. Approximately 124 phytochemicals have been isolated from *CentellaA*, with scientific investigations validating their bioactive efficacy, making them useful in pharmaceutical, cosmetic, and food industries [5]. The total phenolic content of *CentellaA* ranged from 3.42  ±  0.03 to 8.32 ±  0.10 mg GAE/g dry extract, and its antioxidant activity ranged from 75% to 86% [6].

**Figure 1:**
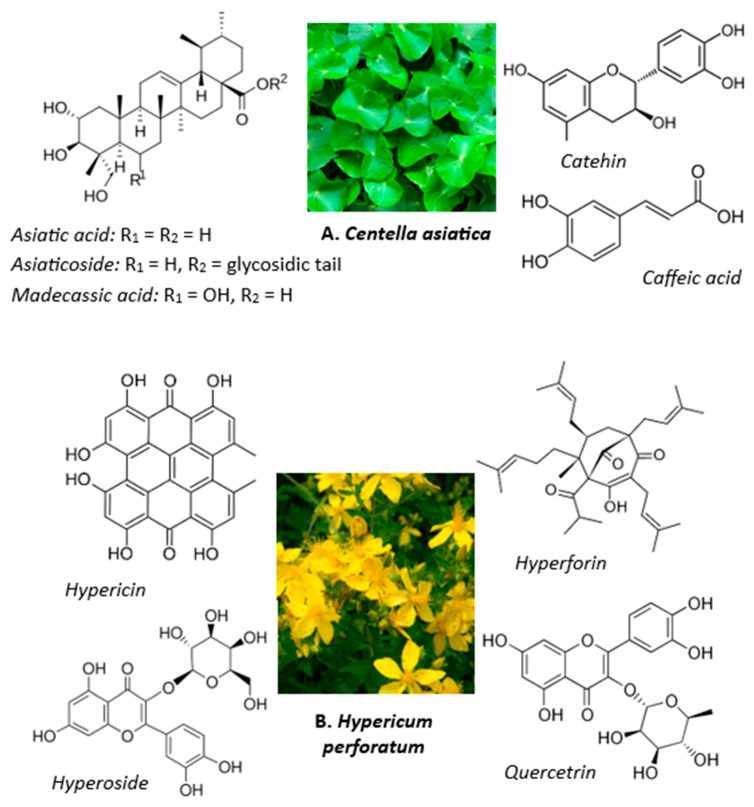
Triterpenes, flavonoids, and other bioactive compounds responsible for the therapeutic effects of *Centella asiatica* (**A**) and *Hypericum perforatum* (**B**).

*Hypericum perforatum* (St. John’s wort) has been used in traditional medicine to treat a wide spectrum of ailments owing to its various bioactive metabolites, including flavonoid glycosides (such as quercetin, hyperoside, quercitrin, isoquercitrin, miquelianin), proanthocyanins and biflavonoids, phloroglucinols (hyperforin), caffeic acid derivatives, and anthocyanidins (Figure 1). Its phytochemical profile is also comprised of naphthodianthrones (hypericin and pseudohypericin), xanthones, and phenylpropanes [7]. Other notable components in *HypericumP* are monoterpenes, sesquiterpenes and their oxygenated derivatives. Flavonoids are the major group of biologically active compounds in *H. perforatum* (ranging from 2% to 5%) and mainly include flavonols (kaempferol and quercetin) and quercetin glycosides (hyperoside, rutin, quercitrin, and isoquercitrin). *HypericumP* plays an important role in the pharmaceutical industry and in modern cosmetology, being one of the most used medicinal plants in the world due to its well-documented pharmacological activity, including antidepressant, antiviral, and antibacterial effects [8]. There are marketed products containing *HypericumP* that are used as phytopharmaceuticals and nutraceuticals [9]. *HypericumP* has been used in skin care products, wound healing and antiaging herbal creams [10,11], but has had the most applications in medicine. Besides its wound healing and anti-inflammatory properties, it has the ability to relieve gastrointestinal and anxiety disorders, as reported by the Committee on Herbal Medicinal Products [12].

Despite the therapeutic benefits in managing various disorders, both *CentellaA* and *HypericumP*, present a series of shortcomings that diminish their reported pharmacological effects, such as cardioprotective, hepatoprotective, neuroprotective actions, antidiabetic, anti-ulcer and antitumor effects, and wound healing properties [13]. Several phytochemical disadvantages of *CentellaA* and *HypericumP* are their high molecular weights, chemical instability, water solubility, and low bioavailability, which hampers skin absorption after topical administration [14]. Therefore, strategies based on colloidal drug delivery systems have been developed for herbal extracts.

Nanostructured lipid carriers (NLC) are multiparticulate colloidal drug delivery systems composed of a binary mix of solid and liquid lipids, stabilized by one or a combination of surfactants used within the range of 1.5% to 5% *w*/*v* [15]. These nanosized lipid-based systems are included in the size range of 50 nm to 500 nm [16], and are designed to encapsulate active compounds, particularly those of natural origin, for sustained release and enhanced bioavailability [17]. NLCs are typically formulated with biocompatible and non-toxic lipids, offering a safe and effective platform for nano-drug delivery [18]. Due to their safety, stability, and high drug loading capacity compared to other lipid-based nanocarriers, NLCs gained the attention of researchers seeking to formulate safe and effective drug carriers, being a perfect alternative to liposomes and microemulsions. In pharmaceutical or cosmetic formulations, active-loaded NLCs act as a sustained-release depot, offering an appropriate release of active compounds. They have the capacity to entrap both hydrophilic and lipophilic candidate drugs, which can minimize tissue irritation and improve the drug’s therapeutic potential [19]. Advanced-nanostructured carriers for accelerated wound healing have been a topic of interest in recent years [20]. The unique properties of NLCs, including their tunable physical and chemical characteristics, offer a precise approach to wound management [21].

Wound healing involves a balance between hemostasis, inflammation, proliferation, and remodeling. Chronic wounds disrupt this balance and present significant clinical challenges that traditional treatments struggle to address. NLCs may offer promising solutions for wound care and may enhance wound healing in various ways [22], including by having antimicrobial properties that combat infection, targeted drug delivery to the wound site, immunomodulation, and promotion of cellular behavior that aids in tissue regeneration [23]. To maximize NLC wound care efficacy, an array of organic-based materials, such as natural polymers, i.e., polysaccharides, proteins, and peptides, can be successfully tailored [24]. For instance, hyaluronic acid (HA) has proven efficacy in bioengineering and biomaterials due to its improved biocompatibility [25], its potential for targeted treatment, and its being also found to have future potential as an immune system modulator [26]. HA is a linear polysaccharide composed of N-acetyl-D-glucosamine and D-glucuronic acid units, with a molecular weight varying from hundreds to millions of Daltons [27]. A literature review revealed extensive research carried out using HA conjugated and/or decorated-NLCs, such as HA-based hesperidin-NLC delivery approach for the management of obesity [28], tacrolimus HA-modified-NLC for cellular uptake and anti-inflammatory activity [29], docetaxel-loaded HA-coated-NLC for in vitro cytotoxicity in breast cancer cells [30], or curcumin loaded into HA-decorated lipid nanocarriers for improved stability and anti-inflammatory effects [31]. To date, only two studies have adopted the solid lipid nanoparticles (SLNs) and the NLC strategy for *CentellaA* entrapping, aiming to confirm antibacterial and anti-inflammatory activity [14,32]. Regarding *HypericumP*, the phytochemical hypericin (from *HypericumP*) was encapsulated in SLN to evaluate phototoxicity and photodynamic efficacy for photodynamic therapy in cancer treatment [33,34], and into NLC for treating vulvovaginal candidiasis caused by *Candida albicans* [35].

Thus, this study investigates for the first time the obtaining and structural characterization of *CentellaA* with *HypericumP* entrapped into NLCs and hybrid hyaluronic acid-NLCs as valuable natural formulations with enhanced bioactivity. As a novel approach that successfully addresses the bioavailability limitations of plant extracts of *CentellaA* with *HypericumP*, we aimed to further emphasize their biological importance by investigating the morphological fluorescence changes induced by NLCs and HA-NLC-*CentellaA* or/and *HypericumP* on the nuclei and skeleton of skin fibroblasts BJ cells. We believed that preserving the main extract bioactives through nano-entrapment would enable us to gain insights into their wound-healing potential. Thus, a study was conducted to evaluate the wound closure effect of scratches on BJ fibroblast cells, which were monitored for 30 h. In addition, we sought to meet the challenge of developing herbal products combining the benefits of herbal extracts and nanotechnology to create value-added herbal products.

## 2. Materials and Methods

### 2.1. Materials

The lipids: glycerol monostearate (GMS) was purchased from Cognis GmbH (Monheim am Rhein, Germany) and shea butter from Solaris Plant SRL (Bucharest, Romania). The milk thistle was purchased from Elemental (Oradea, Romania) with the fatty acids composition of 54.5% linoleic acid (ω-6), 39.67% oleic acid (ω-9), 8.67% palmitic acid, 6.27% methyl stearate. The castor oil was purchased from Solaris Plant (Bucharest, Romania) with fatty acid composition of 91.72% ricinoleic acid, 4.91%, linoleic acid (ω-6), 3.67% oleic acid (ω-9). CentellaA and Hypericum P extracts were procured from S.C. Hofigal Export Import SA, Bucharest, Romania. For the preparation of aqueous extracts from the aerial parts of *CentellaA* and *HypericumP* plants, the ultrasound-assisted extraction method (1 h, 40 °C, 80 kHz, with shaking) was used. The aqueous extracts were pre-frozen at −80 °C, and subsequently lyophilized (Labogene lyophilizer). The amount of polyphenols was 54.8 mg GAE/g lyophilized *CentellaA*, and 322.4 mg GAE/g lyophilized *HypericumP*. The surfactants: soy lecithin (Alfa Aesar), poly(ethylene glycol)-*block*-poly(propylene glycol)-*block*-poly(ethylene glycol)/Poloxamer 188 were purchased from Sigma Aldrich Chemie, and Tween 20 (polyoxyethylenesorbitan monolaurate) was purchased from Merck (Germany). The reactives: NaCl, Na_2_CO_3_ anhydrous, gallic acid, Folin-Ciocâlteu reagent, phosphate buffer (PBS), 6-hydroxy-2,5,7,8-tetramethyl chroman-2-carboxylic acid (Trolox), potassium persulfate, and 2,2-azinobis-(3-ethyl benzthiazoline-6-sulfonic acid) (ABTS), were supplied by Sigma Aldrich Chemie GmbH (Schnelldorf, Germany).

### 2.2. Preparation of Conventional and Hybrid HA-NLC-Entrapping Phytochemical Mixtures

The preparation of NLC phytochemicals and hybrid NLC phytochemicals involved the use of a melt emulsification method coupled with high-pressure homogenization [36,37]. Briefly, a lipid pre-emulsion was formed by contacting under magnetic stirring (70 °C) two phases, a lipid phase containing a mixture of glyceryl monostearate: shea butter: milk thistle oil: castor oil, in a weight ratio of 1:1:0.57:0.28, and an aqueous phase containing a mixture of surfactants of the type polyoxyethylene (20) sorbitan monolaurate: soy lecithin: amphiphilic block copolymer, in a weight ratio of 1:0.29:0.29. Variable amounts of *CentellaA* extract and/or *Hypericum* extract were integrated in the aqueous phase (according to Table 1). The obtained pre-emulsion was maintained at a temperature of 70 °C for 15 min, after which it was subjected to a high shear homogenization step at 12,000 rpm, for 1 min (using a High-Shear Homogenizer PRO250 type, PRO Scientific Inc., Oxford, CT, USA) and subsequently processed through high-pressure homogenization (APV 2000 Lab Homogenizer, Gifhorn, Germany), with six homogenization cycles (196 sec.) at 500 bar. By cooling the nanoemulsion resulting from HPH processing (to room temperature), the lipid nanocarriers were solidified, producing aqueous dispersions of solid NLC-*CentellaA* and/or *HypericumP*.

The blending of NLC with hyaluronic acid with the establishment of electrostatic interactions, hydrophobic attractions, and hydrogen bonds between the amphiphilic and nonionic surfactant shell of NLCs and HA was achieved by dropwise addition and under vigorous stirring a volume of 20 mL HA solution (2 mg/mL) into a volume of 30 mL aqueous dispersion of NLC-*CentellaA* and/or *HypericumP*, in a variable weight ratio specified in Table 1. Lyophilization at −44 °C for 52 h (Martin Christ Alpha 1–2 LD lyophilization system, Germany) produced solid and/or semisolid formulations of polymer–lipid nanocarriers (NLC decorated with HA).

### 2.3. Characterization Methods

#### 2.3.1. Determination of the Size and Physical Stability of NLCs

The evaluation of average diameters (Zave) was carried out using dynamic light scattering (DLS) with a Zetasizer ZS 90 (Malvern Instruments Inc., Malvern, UK), which featured a solid-state laser (670 nm) at a scattering angle of 90° and a temperature of 25.0 ± 0.1 °C. A volume of 100 μL NLC samples was diluted with 25 mL of double-distilled water before the DLS analysis to ensure a reliable light scattering signal. The particle size data were interpreted based on the intensity distribution. Each Zave and PdI value was calculated as the average of three distinct measurements.

The zeta potential (ξ) of the NLCs was determined in a capillary cell utilizing a microelectrophoresis device (Zetasizer Nano ZS, Malvern Instruments Inc., Malvern, UK). Before the analysis, the NLCs were diluted 1:100 with deionized water and adjusted with a 0.9% NaCl solution to prevent multiple scattering effects and to achieve a conductivity of 50 µS/cm.

#### 2.3.2. Differential Scanning Calorimetry Assay

The variations in the crystalline state of the lipid matrix of lyophilized NLCs were analyzed through differential scanning calorimetry (DSC). The thermograms were generated using a differential scanning calorimeter, namely the Jupiter STA 449C (Netzsch, Selb, Germany). The experiment involved weighing 10 mg of free and co-loaded nanocarriers in a standard aluminum pan, with the samples heated at a rate of 5 °C/min, within the temperature range of 20 to 110 °C. An empty aluminum pan was employed as a reference.

#### 2.3.3. ATR-FTIR Spectroscopic Characterization

The infrared spectra of solid/lyophilized NLC- and HA-NLC-herbal extract formulations and vegetal extracts (CentellaA and HypericumP) were recorded with a Perkin-Elmer Spectrum 100 instrument (Perkin-Elmer, Shelton, CT, USA), which was equipped with a horizontal ATR device. FTIR spectra were recorded in the spectral range 4000–600 cm^−1^, with an increment of 4 cm^−1^.

#### 2.3.4. Determination of Entrapment Efficiency

The non-encapsulated *CentellaA* and *HypericumP* extracts were determined by the quantification of polyphenols extracted in water from 0.15 g of lyophilized NLCs. The po-lyphenolic content, expressed in terms of gallic acid equivalents (GAE), was evaluated through the Folin-Ciocâlteu method (ISO 14502-1:2005) [38]. The calibration curve for gallic acid solutions was used in the concentration range 0–100 mg/L of gallic acid (with R^2^ = 0.9919). Each suspension, comprising 0.15 g of lyophilized NLC- and HA-*CentellaA*/NLC-*HypericumP*/NLC-*CentellaA-HypericumP* mixed with 1 mL of water, was gently shaken, subjected to 15,000 rpm for 5 min using a centrifuge (Sigma 2K15, Osterode am Harz, Germany), and subsequently, 0.5 mL of supernatant was collected in a graduated tube. Into the tube was added 2 mL of 10% (*v*/*v*) Folin-Ciocâlteu reagent and 2.5 mL of a 7.5% Na_2_CO_3_ solution, which was homogenized and incubated in a dark room for 1 h. The absorbance of the samples was recorded using a UV–vis spectrophotometer V670 Jasco (Tokyo, Japan), at λ = 765 nm in triplicate. The entrapment efficiency (EE%) of *CentellaA* and *HypericumP* extracts was calculated using the following equations:

(1) $$EE\%=\frac{W_{a}{-W}_{s}}{W_{s}}\times 100$$

where W_a_ is the weight of polyphenols from plant extract added in the nanocarriers, and W_s_ is the analyzed weight of actives in the supernatant.

#### 2.3.5. Evaluation of In Vitro Antioxidant Action

The antioxidant activity of NLC samples and extract was analyzed using the ABTS method, modified by Tincu [39]. A volume of 2 mL each NLC solution (5 mg/mL) and 3 mL of ABTS^●+^ was measured after 4 min, using ethanol as a reference. The measurements were performed using a UV–vis spectrophotometer V670 Jasco (Tokyo, Japan), at λ = 765 nm in triplicate. The blank solutions were prepared identically, by replacing the volume of the NLC with ethanol. The scavenging capacity of ABTS^●+^ was calculated as inhibition (%) using Equation (1):

(2) $$\%\;ABTS\;Inh.=\frac{A_{0}-A_{s}}{A_{0}}\cdot 100$$

where A_0_ is the absorbance of the blank and A_s_ is the absorbance of the sample.

The ABTS analysis was applied for series concentration of NLC samples, and the IC50 value was calculated from polynomial regression between % ABTS inhibition and concentration.

#### 2.3.6. In Vitro Release Study

The in vitro release experiments of polyphenols from NLC extracts, both with and without a surface modifier, were assessed utilizing a Franz diffusion cell (Hanson Research Corporation, Chatsworth, CA, USA).The conditions for the experiment were as follows: 200 µL of NLC sample was placed in the donor chamber on the cellulose membrane (0.1 µm; Whatman, Germany), and the experiments were conducted for 6 h at a temperature of 37 °C. The receptor chamber contained a release medium that was a mixture of ethanol and phosphate-buffered saline at pH 5.5 in a 1:1 ratio. The receptor medium (6 mL) was continuously stirred at 400 rpm and maintained at 37 °C. At specific 1 h time intervals, 0.5 mL of sample was collected from the receptor chamber (for quantitative determinations) simultaneously with the replacement of an identical volume of 0.5 mL receptor medium, ethanol, and PBS mixture = 1:1 (to maintain the constant concentration in the receptor chamber). The concentrations of polyphenolic extracts were determined through UV–vis spectrometry, as described in Section 2.3.4. The absorbance of each sample was recorded at λ = 765 nm.

The release kinetics of polyphenols were calculated using five mathematical models equations: first order: $ln(100-\%RE)=k_{1}t$; Higuchi: $\%RE=k_{2}\sqrt[{2}]{t}$; Hixson–Crowell: $\sqrt[{3}]{100-\%RE}=k_{3}t$, where %RE is the percentage of polyphenols released in time, *t*; *k*_1_, *k*_2_, and *k*_3_ are the rate constants for first order, Higuchi, and Hixson-Crowell, respectively. The value of *n* is the release exponent and could indicate the mechanism of drug release: a value of *n* < 0.43 corresponds to Fickian diffusion; 0.43 < *n* < 0.89 to a non-Fickian transport; a value of *n* > 0.89 is associated to a super case-II transport [40]. The equations corresponding to the models that yielded the highest R^2^ values were chosen as the optimal release kinetic model.

#### 2.3.7. Skin Fibroblast Cells

Human skin fibroblast cells (BJ, ATCC, ^®^ CRL-2522™, Manassas, VA, USA) were used for this study. Cells were cultured in DMEM (Dulbecco’s modified Eagle medium) coated with 10% fetal bovine serum (Gibco), and 100 U/mL penicillin with 100 μg/mL streptomycin (Gibco), as previously described [41]. Cell density varied depending on the experiments, as mentioned below. During the experiments, the cells were grown in a humidified atmosphere of 95% air/5% CO2 at 37 °C. All cell cultivation media and reagents were purchased from Biochrom AG (Gibco, New York, NY, USA).

#### 2.3.8. Cell Viability Assay

For the cell viability assay, 7000 cells were seeded into each well of a 96-well plate. After 24 h, cells were treated for an additional 24 h with various concentrations of the compounds (0, 6.25, 12.5, 25, 50, 100, 200, and 400 µg/mL). Following the desired time of treatment, BJ cell proliferation was assessed using the MTT (Serva, Heidelberg, Germany), applied at a final concentration of 1 mg/mL in each well, and further incubated at 37 °C for 3–4 h. After this time, the medium was removed, and the crystals formed by the metabolically active cells were dissolved in DMSO. The solution absorbance was measured at 570 nm using a Mithras LB 940 plate reader (Berthold Technologies, Bad Wildbad, Germany). Cell viability was calculated relative to untreated controls; all experiments were performed in triplicate and repeated at least three times. Cell viability was determined with the following formula:

% cell viability = corrected absorbance of treated cells/corrected absorbance of control cells × 100

#### 2.3.9. Morphological Evaluation by Fluorescence Microscopy

The morphological changes were investigated by fluorescence microscopy by staining the actin filaments with Phalloidin-FITC (Sigma-Aldrich, Saint Louis, MO, USA), and the nucleus with Hoechst 33,342 (Invitrogen, Karlsruhe, Germany) for the samples where the viability was above 50%. The cells grown on a cover glass in the presence of the samples for 24 h were first washed with phosphate-buffered saline (PBS) three times. Afterwards, the cells were fixed with 4% formaldehyde, rewashed three times with PBS, permeabilized with 0.1% Triton X-100 in PBS, and washed again with PBS three times. Finally, the two fluorescent dyes were added together to the cells and left in the dark, at room temperature, for 1.5 h. In the last step, the cells were washed with PBS three times and fixed with FluorSaveTM (Merck, Darmstadt, Germany). The fluorescence images were taken with a confocal microscope (Andor DSD2 Confocal Unit, Singapore) mounted on an Olympus BX-51 epifluorescence microscope, using a 40× objective. The images were recorded using a DAPI/Hoechst filter cube (excitation filter 390/40 nm, dichroic mirror 405 nm, and emission filter 452/45 nm) in the case of the nucleus and a GFP/FITC filter cube (excitation filter 466/40 nm, dichroic mirror 488 nm, and emission filter 525/54 nm) in the case of actin filaments. The images were further pseudo-colored and overplayed using the ImageJ software (version 1.53a, Madison, WI, USA). Further, the images were analyzed using a MATLAB R2020a routine reported previously to evaluate the cytoskeleton changes [42].

#### 2.3.10. Wound Healing Assay

Cell migration ability of BJ cells treated for 24 h with two concentrations (12.5 and 200 µg/mL) was assessed by the wound healing assay. Cells were grown as a confluent monolayer in the Ibidi culture-insert 2 well, which created a wound with a 500 µm ± 100 µm width. The cells were imaged at baseline and several times (2, 6, 22, 24, and 30 h) after exposure, using an inverted microscope (Olympus) with a 10× objective. Each condition was tested in triplicate, and the experiment was repeated twice on different cell passages. After calculating the wound width as described previously [43], the percentage of wound closure was calculated as previously described [44].

## 3. Results and Discussions

This research presents the development of different NLC and hybrid HA-NLC formulations containing natural lipids in their network core. The choice of lipids in NLC should be made depending upon phytochemical actives, emulsifiers, and lipid compatibility. All NLC formulations (consisting of MSG in combination with milk thistle, castor oil, and shea butter, and a blend of Tw20: soy lecithin: polaxamer as non-ionic and ionic stabilizers), were used to encapsulate 1 and 1.5% of *Centella asiatica* and/or *Hypericum perforatum* as phytochemical actives. The selection of natural lipids was based on their biocompatibility and wide application in eco-friendly cosmetics. Biological assays, including antioxidant activity, release studies, cell viability assays, and wound healing assays, were conducted on optimized HA-NLC to ensure their safety and bioactive effectiveness.

An ideal nanoscale drug-delivery system should provide physical stability over time, high entrapping capacity, herbal active stabilization, and controlled active release. According to our previous and current results, the NLC formulation fulfils all these criteria and contributes to an explicit potentiation of the antioxidant and wound healing properties. A reasonable explanation for this phenomenon might be sought in the presence of polyphenol extracts, including flavonoids and phenolic acids, which play a crucial role in their therapeutic properties.

Wound healing is a complex process, structured in interrelated phases of hemostasis, inflammation, proliferation, and remodeling. Ideally, this mechanism restores tissue integrity, but the process can deviate towards pathologies such as keloid scars. These arise from excessive fibroblast proliferation and disorganized collagen deposition, representing a major challenge in regenerative medicine. As a result, chronic wounds require a personalized treatment to overcome all the abnormalities observed during the healing process: abnormal fibroblast proliferation, inflammation, excessive collagen production or abnormal extracellular matrix (ECM) deposition [45]. To date, different challenges and potential solutions have been reported, such as the use of inhibitors that block fibroblast keloid production in the extracellular matrix by inhibiting STAT3 signaling [46]; an anti-keloid activity by antagonizing the TGF-β/Smad and MAPK/ERK signaling pathways [47]; stem cell therapy, which prevents hypertrophic scar formation and inhibits fibrosis [48]; and RNA therapy that inhibits proliferation of human keloid fibroblasts [49]. However, an innovative and promising approach in managing these conditions is the use of nanocarriers. The results of the present comparative study bring a new formulation as an alternative to those previously reported; new nanoformulations or various proportions thereof may make significant contributions to their activity. Due to their high surface-to-volume ratio, lipid and hybrid nanocarriers maximize the efficiency of the regeneration process, ensuring a targeted release of therapeutic agents directly at the site of injury. The efficiency of these nanocarriers is enhanced by the integration of phytochemicals that possess remarkable anti-inflammatory properties, reduce oxidative stress, and accelerate epithelialization. In the case of keloid scars, phytochemicals can modulate cell signaling pathways, reducing excessive collagen synthesis and preventing the transformation of the wound into a hypertrophic or keloid scar. The synergy between NLC and phytochemicals creates an optimal microenvironment for healing. This combined strategy not only accelerates the closure of acute wounds, but also offers a promising solution for the aesthetic remodeling of fibrotic tissues, transforming the management of complex scars into a more precise and less invasive process.

### 3.1. Size, Stability, and Structural Characterization

Considering the novelty of the HA-based NLC formulations, understanding the role of each component in the structural organization of the hybrid nanostructured carriers is imperative, with a meticulous evaluation of the size and physical stability and structural behavior of various kinds of NLC with/without hyaluronic acid shell. The particle size distribution of NLCs and HA-NLCs depends on the manufacturing process and surfactant–lipids–actives composition. According to the authors of [50], melt emulsification coupled with high-pressure homogenization is an appropriate technique for manufacturing NLCs with main diameters lower than 200 nm. Based on the Z_ave_ and PdI values presented in Table 1, NLCs met the desired specifications. Most NLCs, both with and without hyaluronic acid, showed a PdI value of less than 0.25, indicating that they were homogeneous and had a monodisperse particle size.

A first notable aspect can be observed by comparing the PdI and Zave parameters of unloaded NLCs with and without HA with those of NLCs loaded with *CentellaA/HypericumP* and of NLCs entrapping a blend of two herbal extracts, respectively (Table 1). According to the obtained results, the polydispersity was significantly improved owing to the capture of phytochemical mixtures (e.g., HA-NLCs had values of 195.0 ± 4.02 nm/PdI = 0.303 ± 0.016 while HA-NLC-*CentellaA* had 202.2 ± 1.72/PdI = 0.150 ± 0.01; HA-NLC-*CentellaA-HypericumP I* had 220.3 ± 1.74 nm/PdI = 0.190 ± 0.005), while the size increased slightly after the incorporation of *CentellaA* and/or *HypericumP*. These positive results appear because of the stabilizing role of the phytocompounds in the extracts. In addition, it was demonstrated that polymers such as proteins or polysaccharides are surface-active molecules which can improve the stability of emulsions [51]. The simultaneous co-entrapping of a blend of *CentellaA:HypericumP* = 4:1 (weight ratio) did not impair the host ability of NLCs, as evidenced by the values of Z_ave_ = 221.4 ± 2.08 nm/PdI = 0.224 ± 0.006 of NLC-*CentellaA-HypericumP I*. The same consideration was observed for hybrid HA covering NLC-*CentellaA-HypericumP I*, with Z_ave_ of 220.3 ± 1.74 nm and PdI = 0.190 ± 0.005. However, the increase in the extracts amount resulted in a visible higher average particle diameter, because of the supersaturation of the host capacity of NLCs (Table 1).

The prepared NLCs exhibited long-term physical stability, with an electronegative potential less than—52 mV. Although it was expected that there would be major fluctuations between the surface charge of the NLCs and the HA-coated NLCs, the determination of the electrokinetic potential demonstrated the opposite of what was expected. The surface charges of the NLCs changed imperceptibly and randomly, regardless of the category or amount of herbal extract coopted in the nanocarrier system or the concentration of HA used to coat the NLCs. These results suggest that the HA-adsorbed layer added onto the NLCs may provide a physical barrier against coalescence. In addition, this behavior can also be attributed to the interactions between the polyphenols from extracts and the NLC, since polyphenols may distribute at the oil/tensioactive interface, providing in this way a protective antioxidant effect in aqueous media and on the lipid phase. Similar findings were previously reported by [52] when studying the encapsulation of *CentellaA* in liposomes.

The NLCs showed an excellent polyphenols-loading capacity, with entrapping efficiency values for *CentellaA* and *HypericumP* ranging from 89.5 to 95.3%. According to current results, both formulations, NLC and HA-NLC, fulfil the suitable “host criteria” and can be considered successful delivery systems for hydrophilic herbal extracts. The high upload capacity of the NLCs could be explained by different parameters. The most important is the physical distribution of polyphenols (contained in the *CentellaA* and *Hype-ricumP* extracts) in the hydrophilicity shell of the NLC, as well as the hydrogen bond interactions between the hydrophile phytochemicals and the emulsifier blend, as previously demonstrated [53]. The hybrid formulations containing hyaluronic acid-coated NLCs presented higher %EE values in comparison with conventional NLCs; i.e., the EE% for NLC-*HypericumP* was 93.56% ± 0.04, while the EE determined for HA-NLC-*HypericumP* was 95.26% ± 0.06. In NLC systems with a mix of plant extracts, the efficiency decreased, but insignificantly; for example, EE% = 92.01% ± 0.40 for NLC-herbal extracts and 92.81% ± 0.12 for HA-NLC-herbal extracts.

### 3.2. Thermal Behavior of the NLCs and HA-NLCs That Entrap Herbal Extracts

Analyzing the role of lipid-surfactant herbal extracts in the structural organization of the NLC- and HA-NLC-herbal extract formulations by differential scanning calorimetry (DSC) revealed the thermal properties of the lipid- and hybrid nanocarriers. DSC assays were carried out closely related to the performance of the NLCs and HA-NLCs to integrate into their surfactant shell and/or lipid core the selected herbal extracts, elucidating fundamental requirements for nanocarriers such as: (i) melting temperature (beneficial to verify that lipids remain solid at room temperature and body temperature); (ii) lipid-surfactant-phytochemical interactions between the different components that constitute the lipid nanocarriers (by comparing DSC thermograms of physical blend, NLC-free and NLCs loaded with herbal extracts); (iii) evaluation of the physical stability and crystallinity; less ordered (amorphous) lipid structures are desirable because they offer a superior loading capacity and help prevent the expulsion of the active principle (burst release, often associated with the transition to more ordered crystalline forms). Less crystalline structures have a greater capacity to incorporate the active phytochemicals and favor a controlled and sustained release, avoiding the burst release of the active principle.

**Figure 2:**
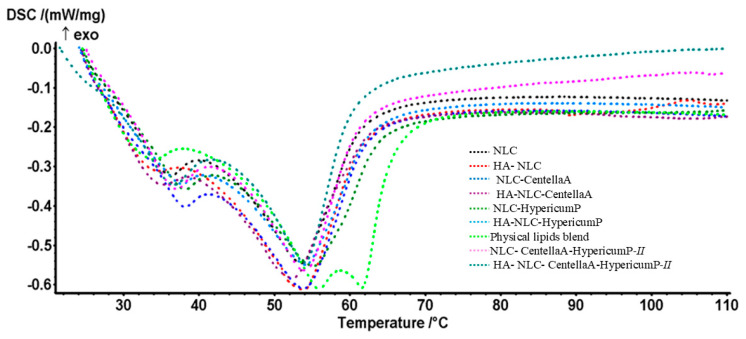
DSC behavior of NLCs and HA-NLCs that entrap *CentellaA* extract and/or *HypericumP* extract.

The DSC behavior analysis was performed for the physical lipid blend, and different NLCs and HA-NLCs, with and without *HypericumP* and or *CentellaA* (Figure 2).

NLCs exhibited typical endothermic peaks related to their various lipid melting points [54]. Shea butter is rich in acylglycerols, but also unsaponifiable fractions (e.g., phytosterols, tocopherols, and triterpenes), so it does not have a single melting point, but a range. The large difference between the melting points of the major fatty acids in the composition of shea butter, for example, stearic acid (69.3 °C) and oleic acid (13–14 °C), present in almost equal proportions [55], explains the existence of the wide endothermic peaks found in Figure 2. The lipids’ calorimetric profile was bimodal, with two endothermic peaks characteristic for shea butter (28 to 45 °C) and mono-, di-, and triacylglycerols (typically 55 ÷ 65 °C). According to the major structural components of the lipid nanocarriers, the thermograms of NLCs exhibited similar bimodal transitions to those of their respective solid lipids, but with melting point shifts of 2 ÷ 4 °C compared to those of a physical lipid blend. Polymorphic profiles often occur in lipid nanocarriers, due to the fraction of polyunsaturated triacylglycerols; in our study, they were assigned to milk thistle oil and castor oil mixture, comprised in the lipid core of NLCs [56].

In NLCs and HA-NLCs, the main contributors that are responsible for their physical and thermal properties comprise acylglycerols, recognized in Figure 2 as a very broad transition centered at 52–54 °C. As expected, the melting point in the NLC thermograms decreased in comparison with the physical lipids blend, due to the high internal disorder of the lipid network of the lipid nanocarriers [57]. This polymorphic NLC behavior contributed to lowering the degree of the lipid matrices’ structural organization, providing an appropriate *CentellaA* and/or *HypericumP* entrapment. All NLCs with herbal extract seemed to improve the thermodynamic properties of the formulations. For instance, the melting point of NLC-*CentellaA/HypericumP* increased approximately 2 °C compared to its free-NLC. The influence of HA coating of NLCs was also visible through decreases in melting points; for example, from 38.1 and 54.9 °C (in NLC-*CentellaA*) to 35.2 °C and 52.9 °C (in HA-NLC-*CentellaA*).

### 3.3. FTIR Characterization of NLCs and HA-NLCs That Entrap CentellaA Extract and/or HypericumP Extract

**Figure 3:**
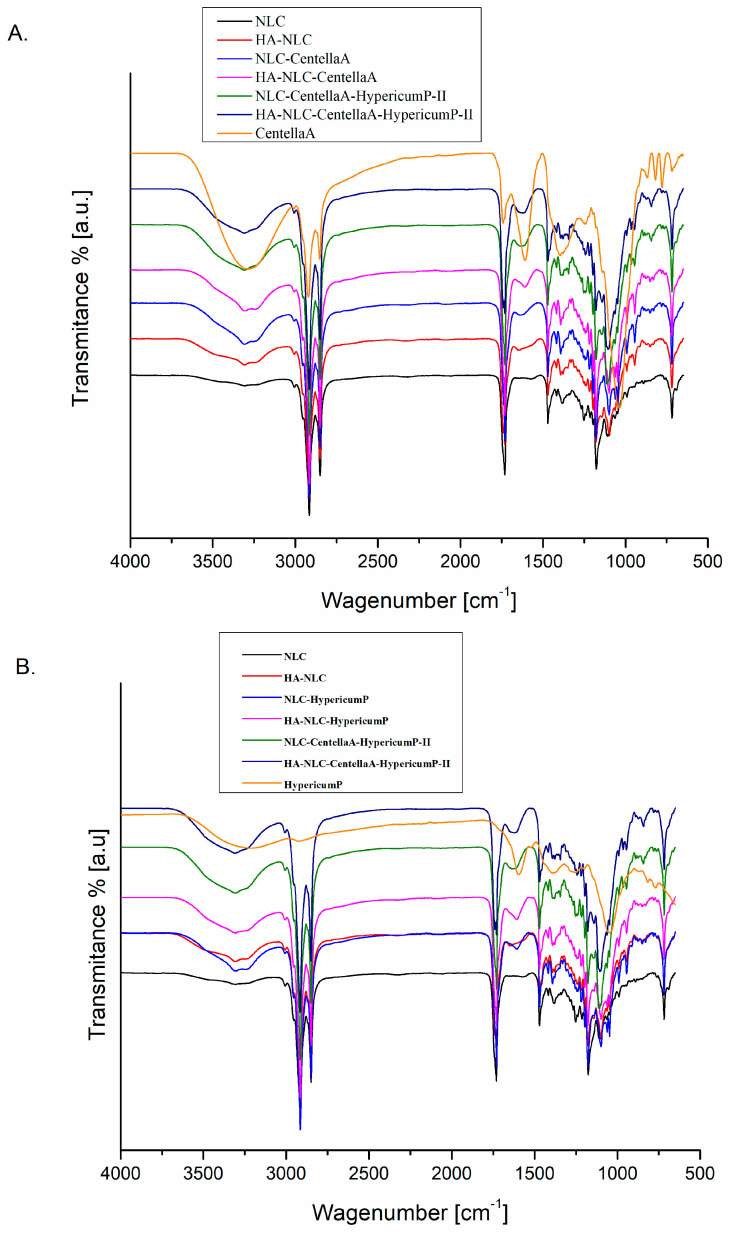
ATR-FTIR spectra of NLCs and HA-NLCs that entrap *CentellaA* extract (**A**) and *HypercumP* extract (**B**) and a mixed system of *CentellaA* and *HypericumP*.

The monitoring of the herbal extract entrapment into NLCs and in NLCs coated with hyaluronic acid, as well as the possible interactions occurring on the surface of the nanocarriers, was carried out by ATR-FTIR. Multiple strong bands were discovered in the IR spectra (Figure 3), many of which were overlaps of the signals arising from the simultaneous presence of the hydroxyl, carboxyl, ester, carbonyl, and amino functional groups belonging to the superficial surfactants, HA and the phytochemicals from *CentellaA* and *HypericumP*. For instance, the distinct bands centered at 2918, 2926, and 2857 cm^−1^, characteristic of the stretching vibration of aliphatic C-H bonds (symmetric and asymmetric), and the wide absorption band at about 3200–3300 cm^−1^, which connects to hydroxyl and amino stretching, are evident. A close look at the peak shape indicates the increased presence of OH and NH groups in NLCs and in HA-NLC-herbal extracts, compared to NLCs without herbal extract. The peak width denotes the extent of hydrogen bonds that occur between -OH, -NH, and other polar groups in phytochemicals, surfactants, and HA. The presence of H bonds is also supported by the slight modification of the frequency of C-OH stretching bonds; i.e., 1377, 1379, 1382, 1384, 1390, and 1395 cm^−1^. These bands corresponding to O-CH and C-OH of the glycosidic region of the polyphenols of *CentellaA* and *HypericumP* extracts were reported by Sotirova in a study of *HypericumP* encapsulation into NLC [58]. In addition, a notable modification of the C-O bond frequency, ~1103 cm^−1^ from NLC and HA-NLC (due to) to 1107 and 1110 cm^−1^ in NLC-*CentellaA-HypericumP* and HA-NLC-*HypericumP*, can also be attributed to the involvement of HO groups in hydrogen bonding [29]. The bands from 1234 cm^−1^ and 1248 cm^−1^ correspond to -OH bending vibration from *CentellaA and HypericumP;* these bands are also recognized in the IR spectra of nanocarriers: 1237 cm^−1^ (NLC-*HypericumP*), 1234 cm^−1^ (HA-NLC-*HypericumP)*, 1238 cm^−1^, and 1243 cm^−1^ in the NLC- and HA-NLC-*CentellaA-HypericumP*, respectively. The strong peak appeared at 1730 cm^−1^ is correlated with the stretching of the carbonyl bond, as reported in other studies [59]. An interesting aspect is the shift by approx. 5 cm^−1^; e.g., 1735 cm^−1^ in NLC versus 1730 cm^−1^ in NLC- and HA-NLC-*CentellaA-HypericumP*.

The infrared spectra of *HypericumP* and *CentellaA* indicate many aromatic rings bearing hydroxyl and carbonyl groups. The aromatic C-H stretching, aliphatic C-H stretching, C=O stretching, and aromatic rings were ascribed to the specific peaks of the HypericumP found at 3200 cm^−1^, 2921 cm^−1^, 2857 cm^−1^, and 1736 cm^−1^, respectively [60]. A representative peak observed in the IR spectra of herbal extracts is the one at 1028 cm^−1^ (from *CentellaA)* and 1060 cm^−1^ (from *HypericumP)*, which are attributed primarily to C-O-H and C-O-C stretching of some oligosaccharides and polysaccharides of herbal extracts and HA [61,62]. C=C–C=C aromatic bonds from polyphenols of *CentellaA* and *HypericumP* are recognized in the region of 1600 cm^−1^ (in *HypericumP)* and 1617 cm^−1^ (in *CentellaA).* In lipid nanocarriers coated with HA and containing *HypericumP* it appeared at 1605 cm^−1^, and in those with *CentellaA* at 1617 cm^−1^, while in nanocarriers that entrapped both herbal extracts, this peak appeared as a broader band centered at 1643 cm^−1^. In a study by Izza, the 1600 cm^−1^ band also corresponds to asymmetric carboxyl groups [63]. The presence of these herbal extracts’ frequencies in all NLC-herbal extracts indicated that the polyphenols were successfully captured predominantly in the outer shell and not inside the lipid core.

### 3.4. Ability of Conventional and HA-Coated NLCs to Manifest Antioxidant Activity

**Figure 4:**
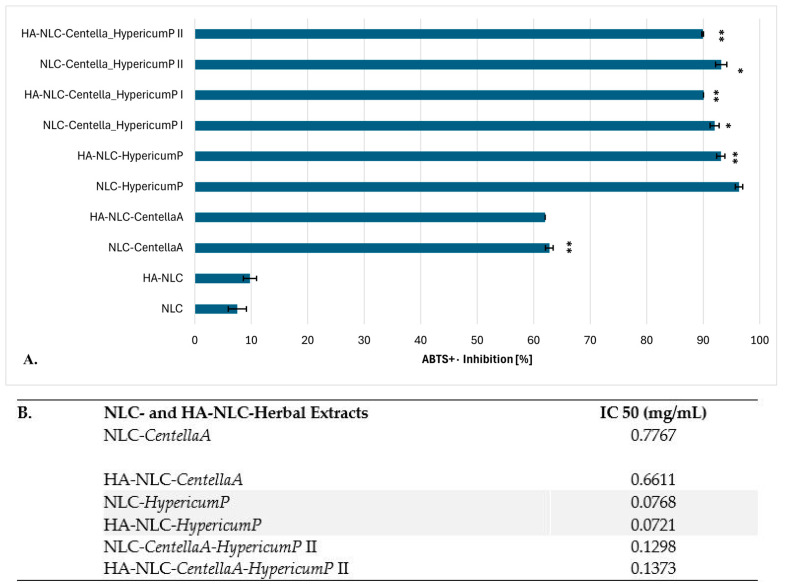
(**A**) Antioxidant capacity of conventional and HA-coated NLCs loaded with *CentellaA/HypericumP* and a blend of *CentellaA* with *HypericumP*. (**B**) IC 50 values determined for selected NLC- and HA-NLC-herbal extracts. * *p* < 0.05; ** *p* < 0.005. Data are expressed as mean ± SD, n = 3 NLC vs. other groups.

The antioxidant activity stems from the NLC- and HA-coated NLC-herbal extracts’ ability to scavenge the free radicals and inhibit lipid peroxidation, a process linked to cellular damage. Although all the tested NLCs possessed a significant ability to counteract the harmful effects of ABTS-type cationic radicals (Figure 4), some differences in the antioxidant capacity were observed, which is expected considering the variation between the main bioactive phytochemicals from the herbal extracts. Current research has shown significant antioxidant effects of *HypericumP* extract in both nanocarrier formulations, the one in which it is captured in NLCs, but also the formulation that comprises both categories, *HypericumP* and *CentellaA.* The most significant values of inhibition of ABTS cationic radicals were 93.13% ± 0.73 and 96.35% ± 0.65, determined for NLC-*HypericumP* and HA-NLC-*HypericumP* (Figure 4A). The primary contributors to the antioxidant activity of *HypericumP* extracts related to the high content of phenolic compounds [64], especially flavonoid compounds (rutin, hyperoside, quercetin) and phenolic acids (chlorogenic acid, caffeic acid). These phenolic compounds have ideal chemical structures for scavenging free radicals (e.g., DPPH, ABTS) and protecting against oxidative stress, playing an important role in both radical scavenging and inhibiting lipid peroxidation activity. According to [65], flavonoid glycosides and phenolic acids, in combination with a sugar unit (e.g., galactoside and rhamnoside), appear to contribute significantly to the neutralization of free radicals, showing high antioxidant activity. As demonstrated by Silva et al. and Orčić et al., the antioxidant potential of *HypericumP* extracts is directly influenced by the phenolics, with quercetin and its glycosidic derivatives being primarily responsible for the antioxidant effect [66,67]. In addition, *HypericumP* has demonstrated activity comparable to or even surpassing that of some standard antioxidant compounds, such as ascorbic acid and synthetic antioxidants [7].

Moderate antioxidant activity was detected for the NLC and HA-NLC formulations containing *CentellaA* extract; the scavenging efficiency of ABTS^●+^ cationic radicals in this case did not exceed 63%. Although *CentellaA* also contains bioactive compounds, including flavonoids, phytosterols, and tannins, which may contribute significantly to its antioxidant effectiveness, its primary active compounds are pentacyclic triterpenoids, such as asiaticoside, madecassoside, Asiatic acid, and madecassic acid, which are responsible for anti-inflammatory and wound-healing properties [13].

The antioxidant structure of extracts and the interactions between them play an important role in the final antioxidant activity. The major antioxidant mechanism of *HypericumP* extract is largely attributed to the ability of these phenolic compounds (flavonoids, phenolic acids) to donate hydrogen atoms to free radicals, stabilizing them. Other mechanisms which contribute to antioxidant efficacy are lipid peroxidation inhibition; i.e., they prevent the oxidation of fats (a key aspect of oxidative damage in biological systems); metal chelation (structurally appropriate hydroxyl groups exhibit a chelating activity of metal ions, which are involved in free radical generation). In addition, compared to *CentellaA, HypericumP* extract contains hypericin and hyperforin, phytocompounds that, although known more for their antidepressant and anti-inflammatory activity, can still contribute to the overall antioxidant profile through a metal ion chelation mechanism [68]. *Hypericum* extract is somewhat more potent, often due to the complexity of the phytochemical profile and the synergistic effect of the compounds. However, there is still insufficient evidence to determine which class of phenols is most responsible for these antioxidant properties.

Results of the antioxidant activity evaluated by the ABTS method expressed as IC_50_ values were achieved on various concentrations intervals; i.e., from 0.02 to 0.75 mg/mL for NLC-*HypericumP* (with an average inhibition of 15.13 to 99.61%), and for the NLC-*CentellaA* it ranged from 0.25 to 2 mg/mL, with an average inhibition of 28.9 to 86.8% (note: concentrations of entire nanocarrier delivery system). The lowest IC_50_ values corresponded to HA-NLC-*HypericumP* and equaled 72.1 ± 0.26 μg/mL, and NLC-*HypericumP* with an IC_50_ value of 76.8 ± 0.63 μg/mL. NLC-*CentellaA* showed the weakest antioxidant activity among the tested nanocarriers.

### 3.5. Phytochemical’s Release from NLC and HA Coated-NLC

**Figure 5:**
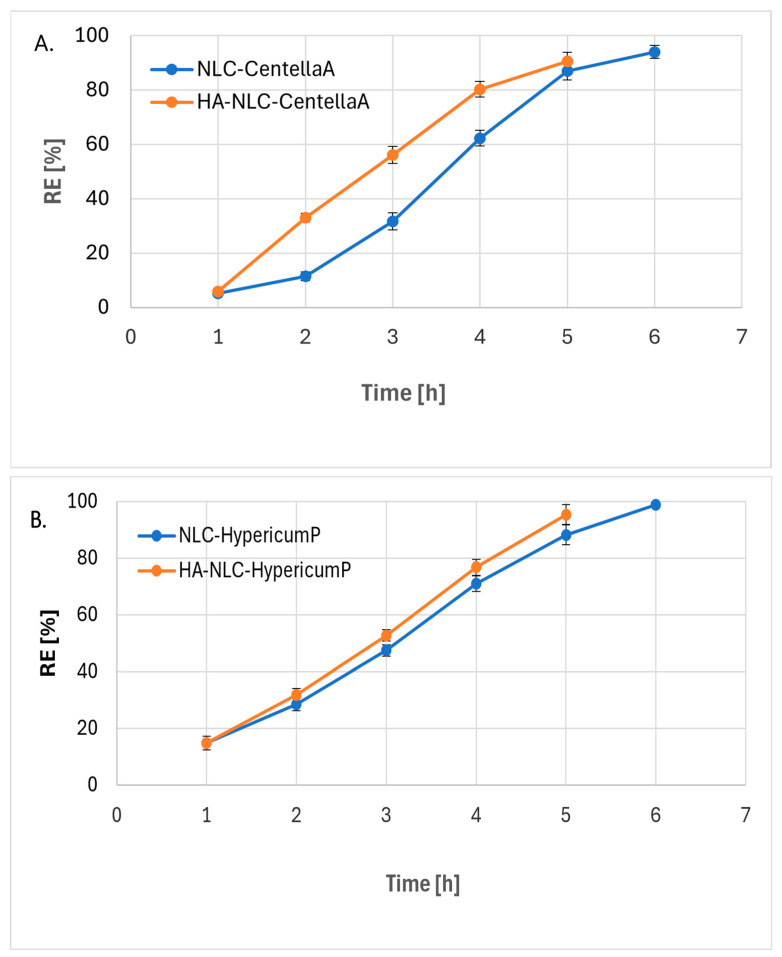

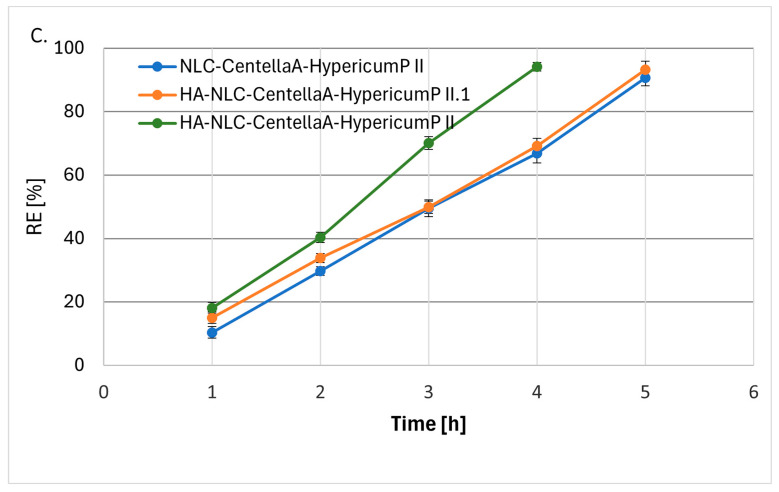
Profiles of in vitro polyphenols release through the Franz cell diffusion method.

In vitro release experiments were performed to evaluate the ability of the NLC and HA-NLC to modulate polyphenols’ delivery using vertical Franz diffusion cells. As can be seen in the comparative results presented in Figure 5, there are significant differences in the release percentages of polyphenols captured in conventional nanocarriers and those coated with hyaluronic acid. NLC formulations with HA enhanced the release rate of polyphenols; e.g., for *CentellaA* captured in HA coated-NLC, 90.65 RE% were determined after 5 h of release experiment versus 86.97% *CentellaA* from conventional NLCs, after 6 h (Figure 5A,B). The faster release from HA-NLCs versus conventional NLCs can be explained by the increased hydrophilicity of the nanocarrier system (due to hyaluronic acid) and, as a result, the easier erosion of the NLC shell, with the release of polyphenols captured in the outer shell.

A relatively similar trend was observed in the case of *HypericumP* extract, with almost total release after 6 h, of 98.9% from HA-NLC-*HypericumP*. For NLC systems with *HypericumP* and mixed ones, which incorporated a blend of *CentellaA* and *HypericumP*, a burst release effect was observed in the first hour (14.18% release from NLC- and HA-NLC-*HypericumP* and between 10.4 and 18.16% from NLC- and HA-NLC-*CentellaA-HypericumP*), followed by a sustained release for the next 5 h (Figure 5B,C). Most likely, the high release percentages detected at the beginning of the release experiment were due to polyphenols that were not trapped in the internal network of surfactants or established extremely weak interactions, easily broken by the acceptor environment.

The NLC systems with mixed herbal extracts content displayed the fastest release period, with a maximum release from HA-NLC-*CentellaA-HypericumP* II of 94.16% polyphenols determined after 4 h of study. This behavior can be attributed to the supersaturation of the spaces available to accommodate both types of herbal mixtures.

Between the two lipid nanocarriers with variable hyaluronic acid coatings (found in a volumetric ratio of aqueous dispersion NLC: HA solution of 1.5:1 and 3:1, Table 1), a significant gap in the release degree was observed, with 70.07% polyphenols released after 3 h from HA-NLC-*CentellaA-HypericumP II*, while HA-NLC-*CentellaA-HypericumP* II.1 reached a threshold of 49.8% polyphenols released. The advanced hydrophilicity of HA-NLC-*CentellaA-HypericumP II*, together with the presence of both plant extracts, is responsible for this behavior.

We have fitted the kinetic curves with different models (Table 2), identifying the best description (R^2^ values were considered) for polyphenols delivery. Most NLC formulations were best fitted by the Peppas-Korsmeyer model (except the HA-NLC-*CentellaA*, which seems to follow a Higuchi-type model), being compatible with an initial burst phase, followed by a delayed release, suggesting Fickian diffusion as the primary release mechanism. In the context of HA-NLC samples, the release rate was higher than that of NLC samples. As previously described by Peleje et al., this behavior is associated with the HA coating, which provides an extra barrier to the diffusion of phytochemicals, leading to a more gradual and controlled release profile [30].

### 3.6. Cytotoxicity Assignment of NLC- and HA-NLC-Entrapping Herbal Extract

**Figure 6:**
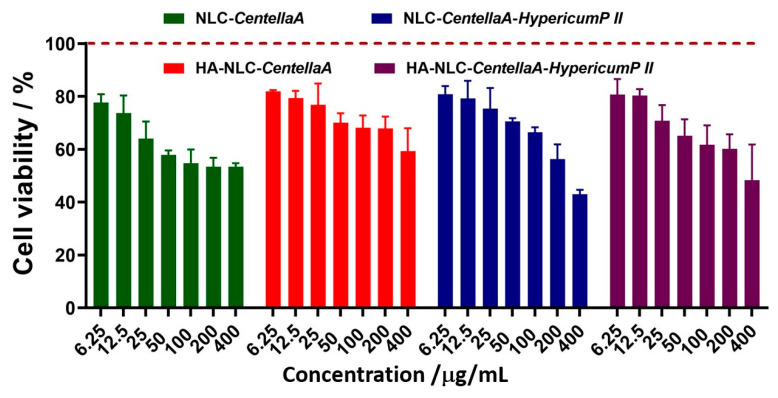
BJ cell viability at 24 h post-treatment with the NLC- and HA-NLC-entrapping *CentellaA* and a blend of *CentellaA* and *HypericumP*.

The cytotoxicity effect induced by increasing concentrations of the NLC-and HA-NLC-*CentellaA* and NLC-and HA-NLC-*CentellaA-HypericumP* was investigated on fibroblast BJ cells. There was no clear dependent trend influenced by the dose and type of *CentellaA* or its association with *HypericumP*, the viability being kept almost at a plateau in several concentrations range (Figure 6). At concentrations of 6.25 and 25 µg/mL, almost all NLCs induce similar effects. Among the tested NLCs, a more profound decrease in cell viability with values lower than 60% and an advanced cytotoxic potential was induced at treatments higher than 50 mg/mL NLC-*CentellaA.* Treatments with concentrations <12.5 mg/mL highlighted for all NLCs safety of use, with the lack of a potential cytotoxic action, with values of approx. 80% cell viability. HA-NLC-*CentellaA* showed a behavior adequate to the intended purpose, being the safest in terms of cell viability and suitable for further tests, while HA-NLC-*CentellaA-HypericumP* showed a moderate dose-dependent toxicity, with a significant decrease in viability for the last concentrations tested.

Despite these results, formulations with NLC coated with hyaluronic acid resulted in visible increases in fibroblast cell viability. This behavior is predictable considering that fibroblasts are cells responsible for the synthesis of some components of the extracellular matrix (ECM), including elastin, collagen, and hyaluronic acid. HA binds to specific receptors on the surface of fibroblasts, most commonly CD44 [25]. According to the study of *Zhang*, this binding initiate intracellular signaling cascades that activate survival and proliferation pathways, such as the protein kinase pathway, MAPK—essential for the processes of mitosis and cell division [26]. Furthermore, HA, being an endogenous structural component of ECM, when present in cell cultures can create a three-dimensional skeleton (scaffold) that mimics the natural environment of fibroblast BJ cells. This biochemical environment optimizes fibroblast adhesion (an essential process for proliferation) and, in the context of wound healing, HA can provide optimal support for fibroblasts BJ cells to migrate to the injured area and multiply [69].

### 3.7. Morphological Evaluation of NLC- and HA-NLC-Herbal Extracts

**Figure 7:**
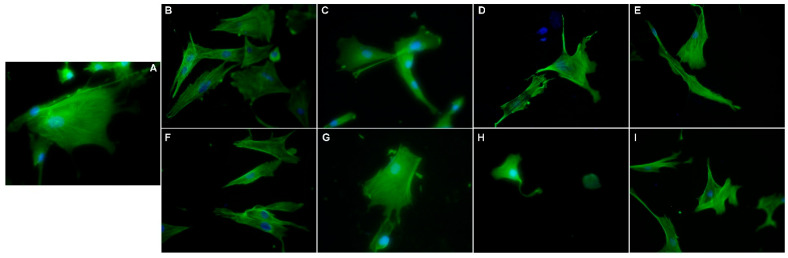
Morphological changes evidenced by fluorescence microscopy: Control (**A**), NLC-*Cente-llaA* ((**B**) 12.5 mM; (**F**) 200 mM), HA-NLC-*CentellaA* ((**C**) 12.5 mM; (**G**) 200 mM), NLC-*CentellaA-HypericumP II* ((**D**) 12.5 mM; (**H**) 200 mM), HA-NLC-*CentellaA-HypericumP II* ((**E**) 12.5 mM; (**I**) 200 mM). Magnification of all images is 40×.

The fluorescence morphological changes induced by the NLC- and HA-NLC-herbal extracts were followed on two different concentrations (12.5 and 200 µg/mL) by analyzing the nuclei and the cytoskeleton. BJ fibroblast cells exhibit the specific fibroblast cells morphology, with an elongated and spindle-shaped cell body and a round or oval nucleus (Figure 7). When analyzing the control condition, the cells grow in an aligned pattern, with a large and granular cytoplasm, with shapes that are mostly bipolar, but some also exhibit a multipolar appearance with pointed ends. According to fluorescence images, a single, protrusive, round to oval and uniformly stained nuclei has been identified in each cell (Figure 7). No obvious fragmentation or condensation was observed, or other abnormalities (like nuclei blebbing or micronuclei), suggesting the nuclei are healthy and intact, and cells appear viable as expected. For the cytoskeleton, particularly the F-Actin filament, long, well-organized filaments with a high polarization along the long axis of the cells can be viewed. The cells are well spread, with some cells exhibiting filopodia-like extensions, which suggests a strong adhesion of the cells to the substrate and active spreading as well as an intact and functional cytoskeleton, with no signs of collapse or retraction. Based on these observations, it can be concluded that the cytoskeleton is characteristic for a viable cell under no obvious stress.

Control cells present a typical healthy BJ fibroblast-like morphology with large and spread-out cells, with prominent actin fibers spanning across the entire cell ending with well-defined lamellipodia (Figure 7A). The nucleus shape is rounded to elongate with no changes. When treated with 12.5 mg/mL NLC-*CentellaA*, the cells look elongated, exhibiting a spindle-shaped morphology, with actin fibers aligned along the axis (Figure 7B). With increasing concentration (to 200 mg/mL NLC-*CentellaA*) the cell becomes more elongated, exhibiting thin extinctions of the actin filaments, indicating a possible reorganization into migratory phenotype (Figure 7F). The nucleus is intact and elongated, for both concentrations.

The BJ cells treated with HA-NLC-*CentellaA* exhibit a morphology closer to control cells (Figure 7C,G), which is correlated with the viability results, indicating biocompatibility with the fibroblast cells. A treatment with NLC-*CentellaA-HypericumP II* obviously affects the fibroblast BJ cells, the cells being smaller and exhibiting a partial cytoskeleton disruption (Figure 7D). A concentration of 200 mg/mL revealed cortical actin filaments, and no stress fibers in the cytosol (Figure 7H). At high concentrations, the cytoskeleton collapses, affecting cell adhesion, which indicates the occurrence of a cytotoxic effect.

For HA-NLC-*CentellaA-HypericumP II*, the cells elongated with thin protrusions and increased polarization of the cytoskeleton (Figure 7E). With increased concentration, the cells exhibited an elongated shape with multiple projections, well defined actin fibers and less organized. These changes suggest a reorganization of the cytoskeleton (Figure 7I). NLC-*CentellaA-HypericumP II* affected the cell morphology the most deeply by depolymerization of the actin fibers; a loss of stress fibers caused a rounded cell with less spreading.

**Figure 8:**
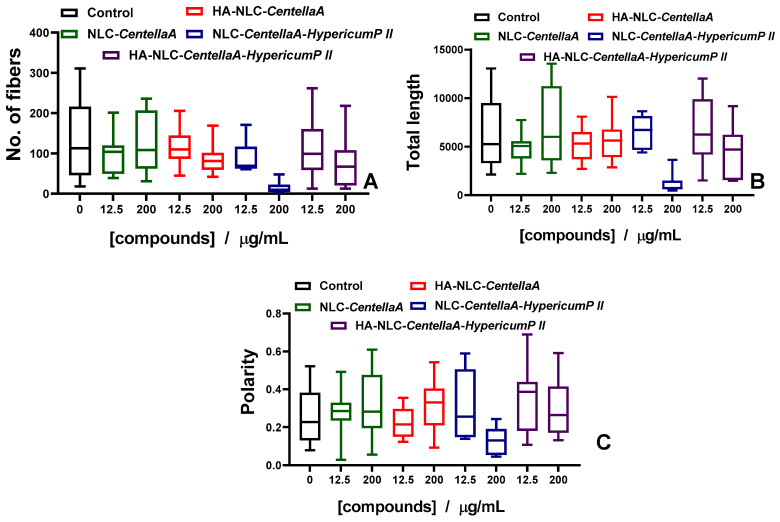
NLC- and HA-NLC-herbal extracts remodel the cytoskeleton of BJ cells. (**A**) Number of fibers, (**B**) total length of fibers, and (**C**) the fibers’ polarity, as determined from fluorescence images using the fiber score algorithm.

The fluorescence images were further analyzed using an adapted MatLab code. Several parameters, such as cytoskeleton fibers number, total length and polarity, are integrated in Figure 8. Upon treatment with NLC-*CentellaA*, the cells retained their intact cytoskeleton, the parameters being only slightly higher. For HA-NLC-*CentellaA*, a decrease in the actin fiber numbers as well as total length was detected; however, the cytoskeleton reorganizes towards a more polarized structure, which may promote migratory phenotype. NLC-*CentellaA-HypericumP II* induces a strong actin depolymerization by reducing the values of all parameters analyzed. HA-NLC-*CentellaA-HypericumP II* has an intermediate disruptor effect, inducing an increase in cytoskeleton polarity despite a reduced number and length of the actin fibers.

**Figure 9:**
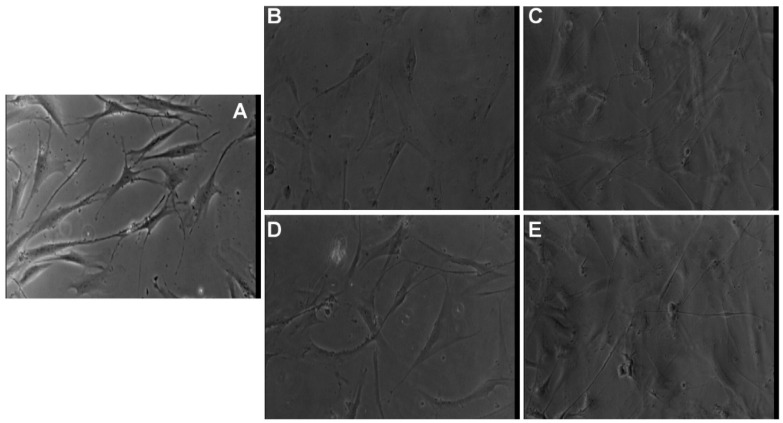
Control (**A**), NLC-*CentellaA* ((**B**), 12.5 μM), HA-NLC-*CentellaA* ((**C**), 12.5 μM), NLC-*CentellaA-HypericumP II* ((**D**), 12.5 μM), HA-NLC-*CentellaA-HypericumP II* ((**E**), 12.5 μM). Magnification of all images is 10×.

**Figure 10:**
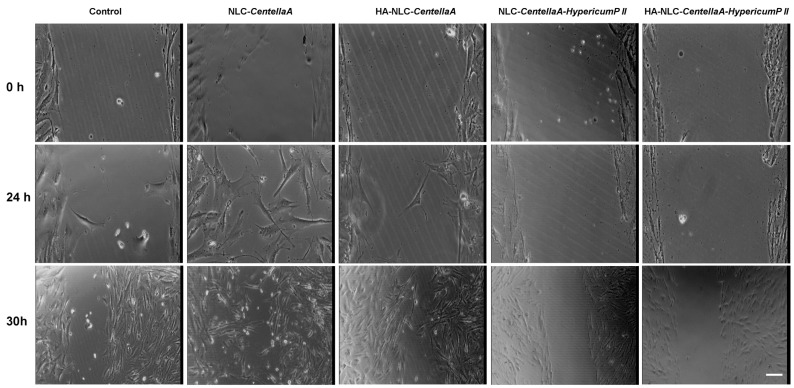
Representative bright field images, with visualization of wound closure, in presence of various types of NLC- and HA-NLC-herbal extracts. The scale bar is the same for all images, with a value of 50 μm.

By analyzing the baseline scratch at time 0 for all conditions, a visible wound gap with a similar cell density along the wound edges was observed (Figure 9). Representative bright-field images presented in Figure 10 show the closure of the wound. After 24 h, for control, cells migrate into the wound, starting to close the gap, with the cells found on the edges presenting a spindle-shaped morphology. For NLC-*CentellaA*-treated cells, migration into the wound is more visible, which suggests an active motility. The presence of HA-NLC-*CentellaA* induces a slower migration compared to NLC-*CentellaA*, and the wound remains more open. A limited cell migration and a wound almost unclosed were observed for NLC-*CentellaA-HypericumP II*, while in the case of HA-NLC-*CentellaA-HypericumP II*, no migrations were detected, and the wound gap remains mostly intact. For a time, a slot of 30 h, the wound closure for control conditions is nearly complete, with confluent cells filling the gap. NLC-*CentellaA* leads also to notable results, the wound closure being almost closed, which indicates strong pro-migratory properties. HA-NLC-*CentellaA-Hype-ricumP* produced an increased migration compared to 24 h; the gap has started closing, but it was not completely closed.

**Figure 11:**
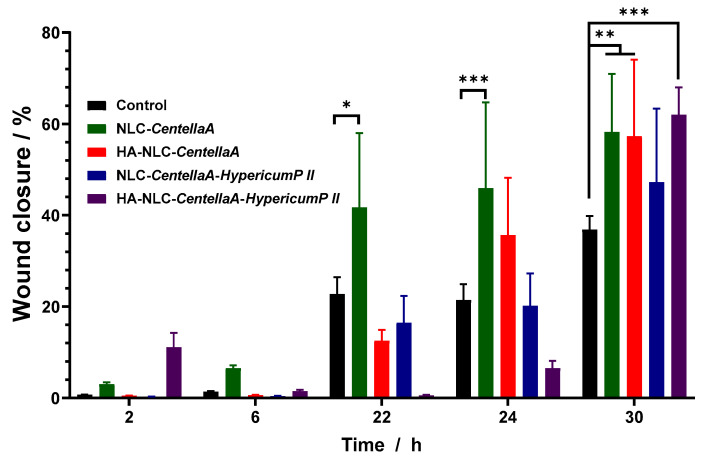
Scratch-wound closure monitored over time in BJ cells, untreated or treated with 12.5 µg/mL of NLCs and HA-NLCs entrapping herbal extract. * *p* < 0.5; ** *p* < 0.01; *** *p* < 0.001; Data are expressed as mean ± SD, n = 3, Control vs. other groups.

The percentage of wound closure is included in Figure 11. By comparing all conditions, it was established that the cells treated with NLC-*CentellaA* exhibited the best wound closure and promoted the fastest migration. This was followed by those treated with HA-NLC-*CentellaA* and HA-NLC-*CentellaA*-*HypericumP II.* Although the evolution of wound healing showed that NLC-CentellaA was better up to 24 h, HA-NLC-CentellaA and HA-NLC-CentellaA-HypericumP II demonstrated similar or better wound closure at 30 h. The hypothesis we can assume is that although NLC-CentellaA maintains healing in the first hours, after 24 h it reaches a plateau that can be overcome by the presence of the other plant extracts and hyaluronic acid.

## 4. Conclusions

Hybrid-NLC-*CentellaA* and/or *HypericumP* extracts are made possible by combining nanotechnology with natural lipids (as a primordial structural skeleton), hyaluronic acid (as an appropriate surface decorating agent for skin regeneration purposes), and natural phytochemical bioactives. Co-loading of a blend of *CentellaA: HypericumP* in a weight ratio of 4:1 and 2:1 did not impair the host ability of NLC, resulting in entrapment efficiency values for *CentellaA* and *HypericumP* ranging from 89.5 to 95.3% and main diameters of 221.4 ± 2.08 nm/PdI = 0.224 ± 0.006 for NLC-*CentellaA-HypericumP* and of 220.3 ± 1.74 nm/PdI = 0.190 ± 0.005 for hybrid HA covering NLC-*CentellaA-HypericumP*. The surface charges of the NLC changed imperceptibly, regardless of the category or amount of herbal extract incorporated into the nanocarrier system or the concentration of HA used to coat the NLC. The zeta potential values, more electronegative than—51 mV, argue that the HA layer adsorption on the NLC provides a physical barrier against coalescence.

The entrapment monitoring of the herbal mixtures in conventional NLC and in HA- coated NLCs, as well as the possible interactions occurring on the surface of the nanocarriers, was carried out by ATR-FTIR and DSC. The polymorphic bimodal DSC profile of NLCs contributed to lower the degree of the lipid core structural organization, while NLCs with herbal extract improved the thermodynamic properties of the formulations. The pre-formulation physicochemical study clarified the role of each evaluated factor influencing the structural-dependent properties of the NLCs and HA-coated-NLCs, allowing selection of the optimized HA-NLC for further biological assays. The NLC formulations displayed an appropriate in vitro release profile, reaching levels of approximately 100% after 6 h of the release study. NLCs coated with HA enhanced the release rate of polyphenols; e.g., for *CentellaA* captured in HA-coated NLCs, 90.65 RE% were determined after 5 h of release experiment versus 86.97% *CentellaA* from conventional NLCs, after 6 h. NLC entrapping both extracts displayed the fastest release period, with a maximum release from HA-NLC-*CentellaA-HypericumP* of 94.16% polyphenols determined after 4 h of release.

NLC- and HA-NLC-herbal extracts proved to be robust antioxidant nanocarriers, as disclosed by the significant ability to counteract the harmful effects of ABTS^+^∙cationic radicals. The antioxidant activity was strongly correlated with the extract’s composition; the most active fractions were those rich in *HypericumP*. The most significant antioxidant values were 93.13% ± 0.73 and 96.35% ± 0.65 determined for NLC-*HypericumP* and HA-NLC-*HypericumP*. The lowest IC_50_ values corresponded to HA-NLC-*HypericumP* and equaled 72.1 ± 0.26 μg/mL, and NLC-*HypericumP* with an IC_50_ value of 76.8 ± 0.63 μg/mL.

The in vitro cytotoxicity revealed that HA-NLC-*CentellaA* was the safest in terms of fibroblast BJ cell viability. NLC coated with HA resulted in visible increases in fibroblast cell viability. It optimized fibroblast adhesion and, in the context of wound healing, may provide optimal support for fibroblast BJ cells to migrate to the injured area and multiply. The morphological fluorescence changes induced by the NLC and HA-NLC-*CentellaA* or/and *HypericumP* on nuclei and skeleton of skin fibroblasts BJ cells were investigated. According to the results obtained, a protrusive and uniformly stained nuclei has been identified in the BJ fibroblast cells, and no fragmentation or condensation or other abnormalities were observed, suggesting the cells appear viable and healthy. The skin cells treated with HA-NLC-*CentellaA* exhibited a morphology closer to control cells, indicating a biocompatibility with the fibroblast BJ cells. Upon treatment with NLCs, the cells retained intact their cytoskeleton, characteristic of a viable cell under no obvious stress, the parameters such as fiber number, total length, and polarity being almost unmodified. Thus, it can be concluded that NLC- and HA-NLC-herbal extracts remodel the cytoskeleton of BJ cells. Representative right-field images showed active and visible motility of NLC-treated cells in the wound area, indicating strong pro-migratory properties. After 24 h, cells began to migrate into the wound and close the wound gap. The evolution of wound healing showed that although NLC-CentellaA is better up to 24 h, HA-NLC-CentellaA and HA-NLC-CentellaA-HypericumP II demonstrated better wound closure at 30 h. NLC-CentellaA maintained healing in the first few hours, but after 24 h reached a plateau that can be overcome by adding HA and the second plant extract.

Overall, this work demonstrates a versatile application of natural lipids and phytochemicals (taking advantage of their diversity, low cost, and abundance), in the development of effective and safe hybrid HA-coated NLCs. As a result of the cumulative results, it can be confidently stated that obtaining these NLCs with HA adaptability to reinforce the skin regeneration and a potent wound healing action represents a desired step for the development of herbal products that meet the challenge of combining the benefits of herbal extracts and nanotechnology to create value-added herbal products.

## Figures and Tables

**Table 1:** Different NLC- and HA-NLC formulations. Size, physical stability, and entrapment efficiency of NLC- and HA-entrapping individual herbal extracts and a blend of *CentellaA* and *HypericumP*.

NLC Formulations *	Herbal Extracts (g)	NLC: HA sol. (mL)	Zave (nm) ± SDS	PdI ± SDS	ξ (mV) ± SDS	EE ± SDS %
NLC	-	-	196.5 ± 2.23	0.226 ± 0.019	−50.7 ± 1.76	-
HA-NLC	-	30:20	195.0 ± 4.02	0.303 ± 0.016	−51.0 ± 1.50	-
NLC-*CentellaA*	1	-	206.1 ± 1.70	0.151 ± 0.013	−54.6 ± 0.66	90.63 ± 0.14
HA-NLC-*CentellaA*	1	30:20	202.2 ± 1.72	0.150 ± 0.011	−58.7 ± 1.27	92.33 ± 0.05
NLC-*HypericumP*	1	-	201.3 ± 2.23	0.176 ± 0.015	−61.7 ± 1.10	93.56 ± 0.04
HA-NLC-*HypericumP*	1	30:20	197.6 ± 1.91	0.163 ± 0.010	−56.9 ± 2.02	95.26 ± 0.06
NLC-CentellaA-*HypericumP I*	0.8 g *CentellaA* + 0.2 g *HypericumP*	-	221.4 ± 2.08	0.224 ± 0.006	−49.8 ± 0.95	92.00 ± 0.40
HA-NLC-*CentellaA-HypericumP I*		30:20	220.3 ± 1.74	0.190 ± 0.005	−52.1 ± 0.46	92.81 ± 0.12
HA-NLC-*CentellaA-HypericumP I.1*		30:10	224.8 ± 0.78	0.211 ± 0.006	−54.4 ± 1.68	92.23 ± 0.36
NLC-*CentellaA-HypericumP II*	1 g *CentellaA* + 0.5 g *HypericumP*	-	197.9 ± 4.06	0.185 ± 0.006	−55.7 ± 0.61	89.52 ± 0.10
HA-NLC-*CentellaA-HypericumP II*		30:20	198.5 ± 0.87	0.213 ± 0.008	−54.9 ± 0.47	90.91 ± 0.05
HA-NLC-*CentellaA-HypericumP II.1*		30:10	193.0 ± 4.59	0.216 ± 0.015	−53.9 ± 0.40	90.52 ± 0.21

* For preparation of 100 g of NLC aqueous dispersions, 2 g emulsifier (1.3 g Tw20 + 0.375 g soy lecithin + 0.375 g polaxamer) and 10 g lipid blend (3.5 g MSG; 3 g milk thistle oil and castor oil; 3.5 g shea butter) were used.

**Table 2:** Kinetic parameters obtained during the in vitro release of polyphenols from NLC and HA-NLC formulations.

NLC and HA-NLC Formulations	Ordin 0		Ordin1		Higuchi		Hixson-Crowell		Peppas-Korsmeyer		
	$\%R=k_{0}t$		$ln\left(100-\%R\right)=k_{1}t$		$\%R=k_{2}\sqrt{t}$		$\sqrt[{3}]{100-\%R}=k_{3}t$		$\%R=k_{4}\sqrt[{n}]{t}$		
	*R* ^2^	*k*_0_	*R* ^2^	*k*_1_	*R* ^2^	*k*_2_	*R* ^2^	*k*_3_	*R* ^2^	*k*_4_	*n*
NLC-*CentellaA*	0.9467	17.728	0.8582	0.4742	0.9030	67.597	0.8745	0.0884	**0.9747**	1.4823	0.2772
HA-NLC-*CentellaA*	0.9778	19.994	0.9231	0.4846	**0.9946**	70.999	0.9464	0.0860	0.9526	1.9841	0.2902
NLC-*HypericumP*	0.9924	17.335	0.8198	0.7928	0.9661	60.650	0.8867	0.0699	**0.9936**	2.6500	0.4432
HA-NLC-*HypericumP*	0.9942	19.524	0.863	0.6893	0.9766	66.226	0.8028	0.1117	**0.9988**	2.683	0.4268
NLC-*CentellaA-HypericumP*	0.9918	18.555	0.8933	0.5340	0.9833	64.428	0.8525	0.0786	**0.9962**	2.3920	0.3727
HA-NLC-*CentellaA-HypericumP* II	0.9931	24.023	0.8916	0.861	0.9827	76.74	0.8476	0.1369	**0.9985**	2.8923	0.4157
HA-NLC-*CentellaA-HypericumP* II.1	0.9945	18.448	0.8407	0.5835	0.9702	61.565	0.7894	0.090	**0.9984**	2.7164	0.4489

## Data Availability

The original contributions presented in this study are included in the article material. Further inquiries can be directed at the corresponding author.

## References

[1] Prathumwon C., Anuchapreeda S., Kiattisin K., Panyajai P., Wichayapreechar P., Surh Y.-J., Ampasavate C. (2025). Curcumin and EGCG combined formulation in nanostructured lipid carriers for anti-ageing applications. Int. J. Pharm. X.

[2] Singh L.S., Singh W.S. (2024). Centella asiatica and its bioactive compounds: A comprehensive approach to managing hyperglycemia and associated disorders. Discov. Plants.

[3] Baljak J., Bogavac M., Karaman M., Srđenović Čonić B., Vučković B., Anačkov G., Kladar N. (2024). Chemical Composition and Biological Activity of Hypericum Species—*H. hirsutum*, *H. barbatum*, *H. rochelii*. Plants.

[4] Gray N.E., Magana A.A., Lak P., Wright K.M., Quinn J., Stevens J.F., Maier C.S., Soumyanath A. (2017). *Centella asiatica*—Phytochemistry and mechanisms of neuroprotection and cognitive enhancement. Phytochem. Rev..

[5] Torbati F.A., Ramezani M., Dehghan R., Amiri M.S., Moghadam A.T., Shakour N., Elyasi S., Sahebkar A., Emami S.A., Barreto G.E., Sahebkar A. (2021). Ethnobotany, Phytochemistry and Pharmacological Features of Centella asiatica: A Comprehensive Review. Pharmacological Properties of Plant-Derived Natural Products and Implications for Human.

[6] Zainal W.N.H.W., Musahib F.R., Zulkeflee N.S. (2019). Comparison of total phenolic contents and antioxidant activities of Centella asiatica extracts obtained by three extraction techniques. Int. J. Eng. Technol. Sci..

[7] Błońska-Sikora E., Zielińska A., Dobros N., Paradowska K., Michalak M. (2025). Polyphenol and Flavonoid Content and Antioxidant Activity of *Hypericum perforatum* L. (St. John’s Wort) Extracts for Potential Pharmaceutical and Cosmetic Applications. Appl. Sci..

[8] Barnes J., Anderson L.A., Phillipson J.D. (2001). St John’s wort (*Hypericum perforatum* L.): A review of its chemistry, pharmacology and clinical properties. J. Pharm. Pharmacol..

[9] Kapoor S., Chandel R., Kaur R., Kumar S., Kumar R., Janghu S., Kaur A., Kumar V. (2023). The flower of *Hypericum perforatum* L.: A traditional source of bioactives for new food and pharmaceutical applications. Biochem. Syst. Ecol..

[10] Algül D., Kılıç E., Özkan F., Uzuner Y.Y. (2025). Wound Healing Effects of New Cream Formulations with Herbal Ingredients. Pharmaceutics.

[11] Aydinli E., Demir B., Goksu H. (2023). Preparation of *Centella asiatica* (L.) and *Hypericum perforatum* (St. John’s Wort) Plant Extracts and Development of Anti-Aging Herbal Cream Formulations. Int. J. Tradit. Complement. Med. Res..

[12] Committee on Herbal Medicinal Products (HMPC), European Union Herbal Monograph on *Hypericum perforatum* L., herba—Revision 1, European Medicines Agency, Amsterdam, The Netherlands, EMA/HMPC/7695/2021. https://www.ema.europa.eu/en/documents/herbal-monograph/final-european-union-herbal-monograph-hypericum-perforatum-l-herba-revision-1_en.pdf.

[13] Diniz L.R.L., Calado L.L., Duarte A.B.S., de Sousa D.P. (2023). *Centella asiatica* and Its Metabolite Asiatic Acid: Wound Healing Effects and Therapeutic Potential. Metabolites.

[14] Rodrigues da Rocha P.B., dos Santos Souza B., Marquez Andrade L., dos Anjos J.L.V., Mendanha S.A., Alonso A., Marreto R.N., Taveira S.F. (2019). Enhanced asiaticoside skin permeation by *Centella asiatica*-loaded lipid nanoparticles: Effects of extract type and study of stratum corneum lipid dynamics. J. Drug Deliv. Sci. Technol..

[15] Lacatusu I., Istrati D., Bordei N., Popescu M., Seciu A.M., Panteli L.M., Badea N. (2020). Synergism of plant extract and vegetable oils-based lipid nanocarriers: Emerging trends in development of advanced cosmetic prototype products. Mater. Sci. Eng. C.

[16] Rajoriya V., Gupta R., Vengurlekar S., Singh U.S. (2024). Nanostructured lipid carriers (NLCs): A promising candidate for lung cancer targeting. Int. J. Pharm..

[17] Lacatusu I., Iordache T.A., Mihaila M., Mihaiescu D.E., Pop A.L., Badea N. (2021). Multifaced role of dual herbal principles loaded-lipid nanocarriers in providing high therapeutic efficacity. Pharmaceutics.

[18] Khan S., Sharma A., Jain V. (2023). An Overview of Nanostructured Lipid Carriers and its Application in Drug Delivery through Different Routes. Adv. Pharm. Bull..

[19] Badalkhani O., Pires P.C., Mohammadi M., Babaie S., Paiva-Santos A.C., Hamishehkar H. (2023). Nanogel containing gamma-oryzanol-loaded nanostructured lipid carriers and TiO_2_/MBBT: A synergistic nanotechnological approach of potent natural antioxidants and nanosized UV filters for skin protection. Pharmaceuticals.

[20] Haririan Y., Elahi A., Shadman-Manesh V., Rezaei H., Mohammadi M., Asefnejad A. (2025). Advanced nanostructured biomaterials for accelerated wound healing: Insights into biological interactions and therapeutic innovations: A comprehensive review. Mater. Des..

[21] Orue G.I., Gainza G., Girbau C., Rodrigo A., Aguirre A., José J., Pedraz J., Igartua M., Hernandez R. (2016). LL37 Loaded nanostructured lipid carriers (NLC): A new strategy for the topical treatment of chronic wounds. Eur. J. Pharm. Biopharm..

[22] Lee H.J., Jeong M., Na Y.G., Kim S.J., Lee H.K., Cho C.W. (2020). An EGF- and curcumin-Co-encapsulated nanostructured lipid carrier accelerates chronic-wound healing in diabetic rats. Molecules.

[23] Wathoni N., Suhandi C., Elamin K.M., Lesmana R., Hasan N., Mohammed A.F.A., El-Rayyes A., Wilar G. (2024). Advancements and Challenges of Nanostructured Lipid Carriers for Wound Healing Applications. Int. J. Nanomed..

[24] Mihaila M., Badea N., Birliga M., Bostan M., Albu Kaya M.G., Lacatusu I. (2025). Ginkgo Biloba and Green Tea Polyphenols Captured into Collagen–Lipid Nanocarriers: A Promising Synergistically Approach for Apoptosis Activation and Tumoral Cell Cycle Arrest. Int. J. Mol. Sci..

[25] Aasy N.K.A., Sallam M.A., Ragab D., Abdelmonsif D.A., Aly R.G., Abdelfattah E.-Z.A., Elkhodairy K.A. (2025). CD44-targeted hyaluronic acid-coated imiquimod lipid nanocapsules foster the efficacy against skin cancer: Attempt to conquer unfavorable side effects. Int. J. Bio. Macromol..

[26] Zhang G., Jiang X., Xia Y., Qi P., Li J., Wang L., Wang Z., Tian X. (2025). Hyaluronic acid-conjugated lipid nanocarriers in advancing cancer therapy: A review. Int. J. Bio. Macromol..

[27] Lee J.Y., Spicer A.P. (2000). Hyaluronan: A multifunctional, megaDalton, stealth molecule. Curr. Opin. Cell Biol..

[28] Gopan G., Jose J., Khot K.B., Bandiwadekar A., Deshpande N.S. (2025). Hyaluronic acid-based hesperidin nanostructured lipid carriers loaded dissolving microneedles: A localized delivery approach loaded for the management of obesity. Int. J. Biol. Macromol..

[29] Garg R., Garg A. (2024). Development and evaluation of hyaluronic acid conjugated tacrolimus-loaded nanostructured lipid carriers using moringa oleifera seed oil as liquid lipid. J. Drug Deliv. Sci. Technol..

[30] Peleje I.R., Di Filippo L.D., Luiz M.T., Capaldi Fortunato G., Portuondo D.L., Freitas da Silva M., Carlos I.Z., Duarte J.L., Chorilli M. (2025). Design and in vitro cytotoxicity of docetaxel-loaded hyaluronic acid-coated nanostructured lipid carriers in breast cancer cells. J. Drug Deliv. Sci. Technol..

[31] Feng J., Wang Z., Huang W., Zhao X., Xu L., Teng C., Li Y. (2025). Hyaluronic acid-decorated lipid nanocarriers as novel vehicles for curcumin: Improved stability, cellular absorption, and anti-inflammatory effects. Food Chem..

[32] Bansal K., Bhati H., Vanshita S., Bajpai M. (2024). Recent insights into therapeutic potential and nanostructured carrier systems of *Centella asiatica*: An evidence-based review. Pharmacol. Res.-Mod. Chin. Med..

[33] Lima A.M., Pizzol C.D., Monteiro F.B.F., Creczynski-Pasa T.B., Andrade G.P., Ribeiro A.O., Perussi J.R. (2013). Hypericin encapsulated in solid lipid nanoparticles: Phototoxicity and photodynamic efficiency. J. Photochem. Photobiol. B.

[34] Youssef T., Fadel M., Fahmy R., Kassab K. (2010). Evaluation of hypericin-loaded solid lipid nanoparticles: Physicochemical properties, photostability and phototoxicity. Pharm. Dev. Technol..

[35] Sato M.R., Oshiro-Junior J.A., Rodero C.F., Boni F.I., Sousa Araújo V.H., Bauab T.M., Nicholas D., Callan J.F., Chorilli M. (2022). Photodynamic therapy-mediated hypericin-loaded nanostructured lipid carriers against vulvovaginal candidiasis. J. Med. Mycol..

[36] Lacatusu I., Badea N., Badea G., Mihaila M., Ott C., Stan R., Meghea A. (2019). Advanced bioactive lipid nanocarriers loaded with natural and synthetic anti-inflammatory actives. Chem. Eng. Sci..

[37] Niculae G., Lacatusu I., Badea N., Oprea O., Meghea A. (2013). Optimization of lipid nanoparticles composition for sunscreen encapsulation. UPB Sci. Bull. Ser. B.

[38] (2005). Determination of Substances Characteristic of Green and Black Tea, Part 1: Content of Total Polyphenols in Tea—Colorimetric Method Using Folin-Ciocalteu Reagent, First Edition.

[39] Tincu R., Mihaila M., Bostan M., Istrati D., Badea N., Lacatusu I. (2024). Hybrid Albumin Decorated Lipid-Nanocarrier-Mediated Delivery of Polyphenol-Rich *Sambucus nigra* L. in a Potential Multiple Antitumoural Therapy. Int. J. Mol. Sci..

[40] Dash S., Murthy P.N., Nath L., Chowdhury P. (2010). Kinetic modelling on drug release from controlled drug delivery systems. Acta Pol. Pharm..

[41] Sowa I., Mołdoch J., Dresler S., Kubrak T., Soluch A., Szczepanek D., Strzemski M., Paduch R., Wójciak M. (2023). Phytochemical Profiling, Antioxidant Activity, and Protective Effect against H_2_O_2_-Induced Oxidative Stress of Carlina vulgaris Extract. Molecules.

[42] Moisă R., Rusu C.M., Deftu A.T., Bacalum M., Radu M., Radu B.M. (2025). Are You a Friend or an Enemy? The Dual Action of Methylglyoxal on Brain Microvascular Endothelial Cells. Int. J. Mol. Sci..

[43] Reyes-Aldasoro C.C., Wilson I., Prise V.E., Barber P.R., Ameer-Beg M., Vojnovic B., Cunningham V.J., Tozer G.M. (2010). Estimation of Apparent Tumor Vascular Permeability from Multiphoton Fluorescence Microscopic Images of P22 Rat Sarcomas In Vivo. Microcirculation.

[44] Grada A., Otero-Vinas M., Prieto-Castrillo F., Obagi Z., Falanga V. (2017). Research Techniques Made Simple: Analysis of Collective Cell Migration Using the Wound Healing Assay. J. Investig. Dermatol.

[45] Kim H.J., Kim Y.H. (2024). Comprehensive Insights into Keloid Pathogenesis and Advanced Therapeutic Strategies. Int. J. Mol. Sci..

[46] McCann K.J., Yadav M., Alishahedani M.E., Freeman A.F., Myles I.A. (2021). Differential responses to folic acid in an established keloid fibroblast cell line are mediated by JAK1/2 and STAT3. Clin. Trial..

[47] Wang W., Qu M., Xu L., Wu X., Gao Z., Gu T., Zhang W., Ding X., Liu W., Chen Y.L. (2016). Sorafenib exerts an anti-keloid activity by antagonizing TGF-β/Smad and MAPK/ERK signaling pathways. J. Mol. Med..

[48] Khaity A., Albakri K., Al-Dardery N.M., Yousef Y.A.S., Foppiani J.A., Lin S.J. (2025). Adipose-Derived Stem Cell Therapy in Hypertrophic and Keloid Scars: A Systematic Review of Experimental Studies. Plast. Surg..

[49] Cai Y., Yang W., Pan M., Wang C., Wu W., Zhu S. (2018). Wnt2 knock down by RNAi inhibits the proliferation of in vitro-cultured human keloid fibroblasts. Medicine.

[50] Ott C., Lacatusu I., Badea G., Grafu I.A., Istrati D., Babeanu N., Stan R., Badea N., Meghea A. (2015). Exploitation of amaranth oil fractions enriched in squalene for dual delivery of hydrophilic and lipophilic actives. Ind. Crops Prod..

[51] Tamang N., Shrestha P., Khadka B., Mondal M.H., Saha B., Bhattarai A. (2022). A Review of Biopolymers’ Utility as Emulsion Stabilizers. Polymers.

[52] Tripathy S., Srivastav P.P. (2023). Encapsulation of *Centella asiatica* leaf extract in liposome: Study on structural stability, degradation kinetics and fate of bioactive compounds during storage. Food Chem. Adv..

[53] Lacatusu I., Badea N., Badea G., Brasoveanu L., Stan R., Ott C., Oprea O., Meghea A. (2016). Ivy leaves extract based—Lipid nanocarriers and their bioefficacy on antioxidant and antitumor activities. RSC Adv..

[54] Kumar R., Singh A., Sharma K., Dhasmana D., Garg N., Siril F.P. (2020). Preparation, characterization and in vitro cytotoxicity of fenofibrate and nabumetone loaded solid lipid nanoparticles. Mater. Sci. Eng. C.

[55] Bunjes H., Unruh T. (2007). Characterization of lipid nanoparticles by differential scanning calorimetry, X-ray and neutron scattering. Adv. Drug Deliv. Rev..

[56] Soleimanian Y., Goli S.A.H., Varshosaz J., Sahafi S.M. (2018). Formulation and characterization of novel nanostructured lipid carriers made from beeswax, propolis wax and pomegranate seed oil. Food Chem..

[57] Zielinska A., da Ana R., Fonseca J., Szalata M., Wielgus K., Fathi F., Oliveira M.B.P.P., Staszewski R., Karczewski J., Souto E.B. (2023). Phytocannabinoids: Chromatographic Screening of Cannabinoids and Loading into Lipid Nanoparticles. Molecules.

[58] Sotirova Y., Gugleva V., Stoeva S., Kolev I., Nikolova R., Marudova M., Nikolova K., Kiselova-Kaneva Y., Hristova M., Andonova V. (2023). Bigel Formulations of Nanoencapsulated St. John’s Wort Extract—An Approach for Enhanced Wound Healing. Gels.

[59] Zhua J., Rena Z., Zhang G., Guoa X., Ma D. (2006). Comparative study of the H-bond and FTIR spectra between 2,2-hydroxymethyl propionic acid and 2,2-hydroxymethyl butanoic acid. Spectrochim. Acta A Mol. Biomol. Spectrosc..

[60] Rey-Méndez R., Rodríguez-Argüelles M.C., González-Ballesteros N. (2022). Flower, stem, and leaf extracts from *Hypericum perforatum* L. to synthesize gold nanoparticles: Effectiveness and antioxidant activity. Surf. Interfaces.

[61] Soleimanifar M., Jafari S.M., Assadpour E. (2020). Encapsulation of olive leaf phenolics within electrosprayed whey protein nanoparticles; production and characterization. Food Hydrocoll..

[62] Geetha N., Harini K., Joseph M., Sangeetha R., Venkatachalam P. (2019). A Comparison of microwave assisted medicinal plant extractions for detection of their phyto-compounds through qualitative phytochemical and FTIR analyses. Iran. J. Sci. Technol. Trans. A Sci..

[63] Izza N., Watanabe N., Okamoto Y., Wibisono Y., Umakoshi H. (2022). Characterization of entrapment behavior of polyphenols in nanostructured lipid carriers and its effect on their antioxidative activity. J. Biosci. Bioeng..

[64] Sotirova Y., Ivanova N., Ermenlieva N., Vilhelmova-Ilieva N., Simeonova L., Metodiev M., Gugleva V., Andonova V. (2025). Antimicrobial and Antiherpetic Properties of Nanoencapsulated *Hypericum perforatum* Extract. Pharmaceuticals.

[65] Hassanpour S.H., Doroudi A. (2023). Review of the antioxidant potential of flavonoids as a subgroup of polyphenols and partial substitute for synthetic antioxidants. Avicenna J. Phytomed..

[66] Silva B.A., Ferreres F., Malva J.O., Dias A.C.P. (2005). Phytochemical and antioxidant characterization of *Hypericum perforatum* alcoholic extracts. Food Chem..

[67] Orčić D.Z., Mimica-Dukić N.M., Francišković M.M., Petrović S.S., Jovin E.Đ. (2011). Antioxidant activity relationship of phenolic compounds in *Hypericum perforatum* L.. Chem. Cent. J..

[68] Alahmad A., Alghoraibi I., Zein R., Kraft S., Dräger G., Walter J.-G., Scheper T. (2022). Identification of Major Constituents of *Hypericum perforatum* L. Extracts in Syria by Development of a Rapid, Simple, and Reproducible HPLC-ESI-Q-TOF MS Analysis and Their Antioxidant Activities. ACS Omega.

[69] Gagneja S., Capalash N., Sharma P. (2024). Hyaluronic acid as a tumor progression agent and a potential chemotherapeutic biomolecule against cancer: A review on its dual role. Int. J. Biol. Macromol..

